# Drought Impacts on Plant–Soil Carbon Allocation—Integrating Future Mean Climatic Conditions

**DOI:** 10.1111/gcb.70070

**Published:** 2025-02-12

**Authors:** Vinzent Leyrer, Juliette Blum, Sven Marhan, Ellen Kandeler, Telse Zimmermann, Bernd J. Berauer, Andreas H. Schweiger, Alberto Canarini, Andreas Richter, Christian Poll

**Affiliations:** ^1^ Department of Soil Biology, Institute of Soil Science and Land Evaluation University of Hohenheim Stuttgart Germany; ^2^ Department of Agronomy, Institute of Crop Science University of Hohenheim Stuttgart Germany; ^3^ Department of Plant Ecology, Institute of Landscape and Plant Ecology University of Hohenheim Stuttgart Germany; ^4^ Centre for Microbiology and Environmental Systems Science University of Vienna Vienna Austria; ^5^ Department of Biological, Geological, and Environmental Sciences (BiGeA) Alma Mater Studiorum—University of Bologna Bologna Italy

**Keywords:** climate change, drought, legacy effect, mean climatic conditions, microbial activity, rewetting, temperate arable soil

## Abstract

Droughts affect soil microbial abundance and functions—key parameters of plant–soil carbon (C) allocation dynamics. However, the impact of drought may be modified by the mean climatic conditions to which the soil microbiome has previously been exposed. In a future warmer and drier world, effects of drought may therefore differ from those observed in studies that simulate drought under current climatic conditions. To investigate this, we used the field experiment ‘Hohenheim Climate Change,’ an arable field where predicted drier and warmer mean climatic conditions had been simulated for 12 years. In April 2021, we exposed this agroecosystem to 8 weeks of drought with subsequent rewetting. Before drought, at peak drought, and after rewetting, we pulse‐labelled winter wheat in situ with ^13^CO_2_ to trace recently assimilated C from plants to soil microorganisms and back to the atmosphere. Severe drought decreased soil respiration (−35%) and abundance of gram‐positive bacteria (−15%) but had no effect on gram‐negative bacteria, fungi, and total microbial biomass C. This pattern was not affected by the mean precipitation regime to which the microbes had been pre‐exposed. Reduced mean precipitation had, however, a legacy effect by decreasing the proportion of recently assimilated C allocated to the microbial biomass C pool (−50%). Apart from that, continuous soil warming was an important driver of C fluxes throughout our experiment, increasing plant biomass, root sugar concentration, labile C, and respiration. Warming also shifted microorganisms toward utilizing soil organic matter as a C source instead of recently assimilated compounds. Our study found that moderate shifts in mean precipitation patterns can impose a legacy on how plant‐derived C is allocated in the microbial biomass of a temperate agroecosystem during drought. The overarching effect of soil warming, however, suggests that how temperate agroecosystems respond to drought will mainly be affected by future temperature increases.

## Graphical abstract

The flow of carbon from plants to roots to the soil microbial biomass and back to the atmosphere is a key process in terrestrial ecosystems. This process is highly affected by drought. In the future, however, the overall climatic conditions under which drought occurs will be different from today. It will be warmer and drier. The study shows that in a warmer and drier future, drought will affect the flow of carbon differently than it does today, mainly because of the increase in temperature in a future warmer world.

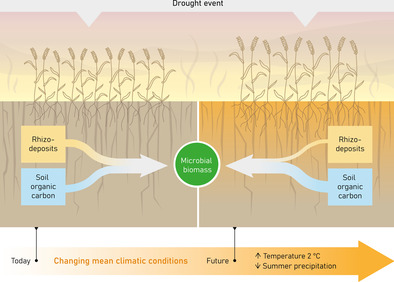

## 1 Introduction

The two main processes driving carbon (C) fluxes between terrestrial ecosystems and the atmosphere are photosynthesis and soil respiration (Hashimoto, Ito, and Nishina 2023). Carbon assimilated during photosynthesis is partitioned into different above‐ and belowground compartments, respired, or exuded at the root‐soil interface via rhizodeposition. In soils, plant‐derived organic can then be either assimilated and invested into anabolic reactions by microbes (i.e., growth) or respired as part of their energy metabolism. The rate of these microbial processes, elemental within terrestrial C cycling, is largely controlled by precipitation and temperature and therefore vulnerable to drought (Xia et al. 2023). Consequently, drought affecting microbial abundance and activity patterns has major implications for soil element cycling, the basis of ecosystem functions and services. It is thus of interest to understand how more frequent and intense droughts in temperate climates (Calvin et al. 2023) will affect soil C cycling. However, predicting future drought impacts on plant–soil C cycling based on studies that observe the direct effects of drought may miss a crucial aspect.

In realistic future scenarios, not only will droughts become more frequent, but also the mean climatic conditions under which they occur will become drier (Drobinski et al. 2020), with decreasing average summer precipitation (Tuel and Eltahir 2021). This should be taken into account, as even moderate reductions in precipitation can lead to shifts in microbial communities and functions (Zhao et al. 2016). From the perspective of ecological theory, such adaptational shifts in response to changes in environmental conditions may leave an imprint on the system's future response to a stressor like drought; this is defined as a ‘legacy effect’ (Cuddington 2011). Legacy effects can result from pulse stressors such as drought (Vilonen et al. 2023) to more constant stressors such as land‐use change (Bürgi, Östlund, and Mladenoff 2017). Accordingly, drought impacts on C allocation may therefore depend on the system's previous exposure to water availability. Consequently, plant–soil C allocation during future droughts may differ from patterns we observe today.

Legacy effects of water scarcity on the composition of soil microbial communities and their physiological traits have led to changes in plant–soil interactions (Kaisermann et al. 2017; Buchenau, van Kleunen, and Wilschut 2022), root morphology (Lozano et al. 2022), and rhizodeposition (Nannipieri et al. 2023). These studies explored legacy effects in ecosystems with perennial vegetation, but we are not aware of any study testing these effects in annually cropped agroecosystems. In arable soils, root‐dependent C input is particularly important for C cycling dynamics due to the removal of aboveground biomass during harvest (Poeplau, Don, and Schneider 2021). This characteristic may provide unique insights into microbially mediated legacy effects because the use of annually sown seeds, produced under ambient climatic conditions, disconnects them from legacies occurring in perennial vegetation.

During drought, important microbial‐mediated processes like nutrient cycling and decomposition are interrupted as soil bacteria and fungi are forced to switch their metabolism to survival strategies. They become spatially disconnected from essential resources and must adjust their osmotic potential to meet the external water potential (Schimel 2018). Consequently, microbial resource acquisition is significantly altered or ceases altogether when soil microorganisms temporarily enter dormancy (Salazar, Lennon, and Dukes 2019). In addition, rewetting after drought is highly stressful for microorganisms as they must quickly adapt their internal solute potential to prevent cell burst (Schimel 2018). The importance of soil C cycling by rewetting dry soils is seen in large CO_2_ pulses, the ‘Birch effect’ (Birch 1958); these pulses contribute a significant fraction of the annual CO_2_ release from soils to the atmosphere (Kim et al. 2012). Proposed direct drought effects have also been observed to impact microbial C incorporation and turnover during a subsequent drought and rewetting event (Fuchslueger et al. 2016). In a field study, Canarini et al. (2021) found that repeated exposure to extreme water scarcity led to the formation of an ‘ecological memory’ driving both microbial community structure and functions during drought. Overall, studies exploring the legacy of repeated water scarcity suggest that microbial communities that are frequently exposed to water stress are more efficient at using available C to build biomass during drought than communities in long‐term wetter soils (Göransson et al. 2013; Canarini et al. 2021; Leizeaga et al. 2021). Conversely, lower biomass building efficiency in historically wetter soils may result in higher respiration activity. Using soils from a precipitation gradient, researchers found that microbes in historically wetter soils had higher respiration rates in response to moisture changes than those in drier soils (Göransson et al. 2013; Hawkes et al. 2017; Hawkes, Shinada, and Kivlin 2020). However, it is not yet clear whether repeated intense drought and rewetting events may affect a system's response to future drought differently, as opposed to a history of reduced mean summer precipitation. In addition, warming is a key driver of an ecosystem's C flux as it accelerates microbially mediated decomposition (Crowther et al. 2016) but also stimulates biomass accumulation (Lin, Xia, and Wan 2010). The predicted future rise in temperature could, therefore, further affect the system's response to drought and importantly, modify the legacy of reduced summer precipitation.

In this study, we investigated how plant–soil C allocation dynamics will respond to drought and subsequent rewetting under simulated future conditions. Specifically, we asked how soil microorganisms differ in their response to drought in an agroecosystem that has been exposed to long‐term warmer and drier mean climatic conditions compared to a system under ambient temperature and precipitation conditions. We used the ‘Hohenheim Climate Change Field Experiment’ (HoCC) as a research platform (Poll et al. 2013). For 12 years, plots in this arable field were exposed to year‐round warming and reduced summer precipitation to simulate a realistic scenario of mean climatic conditions for the temperate climate in the year 2100. We conducted three ^13^CO_2_ pulse labelling experiments: before the onset of drought, at peak drought, and 2 days after rewetting. This made it possible to identify potential legacy effects of the altered mean precipitation patterns on the microbial use of plant‐derived C. We hypothesized that a microbial community exposed to reduced mean precipitation incorporates plant‐derived C more efficiently during drought, resulting in higher microbial biomass at peak drought compared to the control. Furthermore, we hypothesized that the respiration response to rewetting would be stronger in historically wetter soils compared to drier soils. In addition, warming continued during the drought as we expected warming to interfere with the legacy effects of altered mean precipitation patterns.

## 2 Materials and Methods

### 2.1 Site Description; Long‐Term Warming and Precipitation Treatments

The study was conducted at the ‘Hohenheim Climate Change Experiment’ (HoCC), an arable field at the Heidfeldhof experimental station (University of Hohenheim, Stuttgart, Germany, 48°42′50″ N, 9°11′26″ E, 395 m a.s.l.) (Poll et al. 2013). The soil is a loess‐derived stagnic Luvisol with a silty loam texture (9% sand, 69% silt, and 22% clay), and the site is characterized by mean annual precipitation of 689 mm and a temperature of 10.1°C (1990–2020). In the HoCC experiment, mean precipitation and temperature had been factorially manipulated since 2009 to simulate a future climate scenario predicted for the temperate climate zone until the year 2100 (Umweltbundesamt 2006). Soil was permanently warmed by, on average, 2.1°C with heating cables placed on the soil surface and temperature measurement at 4 cm soil depth (*Ta*: ambient temperature; *Te*: elevated temperature) (Leyrer et al. 2024). Dummy cables were placed on the surface of non‐heated plots as experimental controls. Soil warming was applied year‐round from July 2008 until the end of 2021.

Simulation of drier summer months was achieved using transparent foils on roof constructions, with which precipitation was seasonally manipulated from the beginning of June to the end of August. Precipitation treatments were: (1) ambient precipitation, (2) reduced precipitation amount (−25%), (3) reduced precipitation frequency (−50%), and (4) the combination of reduced precipitation amount (2) and reduced precipitation frequency (3). During the rest of the year (September to May), all soils were exposed to naturally occurring rainfall.

The HoCC experiment was set up in a split‐plot design with four blocks, each block consisting of four main plots. Every main plot was divided into four sub‐plots (1 m × 1 m) with the four precipitation treatments. PVC barriers to a depth of 0.5 m were installed to avoid lateral water movement between sub‐plots and the surrounding soil. Throughout the years, soil temperature and moisture were monitored via temperature probes at 4, 15, and 30 cm soil depths and time domain reflectometry (TDR) probes at depths of 0–15 and 15–30 cm. The HoCC agroecosystem was managed conventionally with manual tillage to a soil depth of 0–30 cm and a crop rotation of spring/winter wheat, spring barley, and winter oilseed rape. More details on crops are given in Drebenstedt et al. (2023). The area between the plots was managed with the same crops as the experimental plots to reduce boundary effects and to achieve micro‐environmental conditions similar to a regular crop stand.

The seasonal precipitation manipulation was carried out from 2009 until 2018. In June 2019 we used the HoCC field experiment for a first drought experiment on this site where all plots were exposed to a drought period of 4 weeks (Leyrer et al. 2022), after which (July and August 2019 and June to August 2020) we continued the application of the previously described precipitation treatments (Table 1).

**TABLE 1:** Overview of the treatments in the HoCC Experiment throughout the years. The regular summer precipitation treatments applied from June to August were: Pa: Ambient precipitation and Pr: Reduced precipitation. These were carried out from 2008 to 2018, July and August 2019, and June to August 2020. The treatment combined a reduction of both the amount and frequency of precipitation (−50% frequency and −25% of the resulting cumulative amount). Soil warming was applied year‐round from the beginning of 2009 to the end of 2021. In 2019, the field experiment was used for a first drought experiment (Leyrer et al. 2022). In 2021, the ^13^C drought experiment was conducted, which is the experiment presented here.

Year(s)	Jan–Apr	May	Jun	Jul	Aug	Sep–Dec
2009–2021	Soil warming					
2009–2018	Ambient		Pa; Pr			Ambient
2019	Ambient		Drought^a^	Pa; Pr		Ambient
2020	Ambient		Pa; Pr			Ambient
2021	Ambient	^13^C drought		Ambient		

^a^ Leyrer et al. (2022).

### 2.2 ^13^C Drought Experiment

For the drought study we used the sub‐plots (1), representing ambient precipitation (from now on referred to as *Pa*), and (4), representing combined reduction of precipitation amount and frequency (from now on referred to as *Pr*). Precipitation treatments were factorially combined with soil warming (*Ta*: ambient temperature; *Te*: elevated temperature), for a total of four treatments.

At the end of April 2021, 8 months after the last application of the regular precipitation treatments, we conducted the drought experiment (Table 1). Beginning April 26, 2021, plots were exposed to a drought period for 58 days by excluding all precipitation via the transparent foiled roof constructions. Drought stress was monitored with Scholander pressure chamber measurements (PMS Instruments Model 1500, Albany, USA) of leaf water potential. Specifically, the youngest, fully developed leaf without visual damage was used for measurement as described in Rodriguez‐Dominguez et al. (2022). The duration of the drought lasted until plants showed severe drought stress (< −1 MPa pre‐dawn). For rewetting, 25 mm of water was applied manually to each sub‐plot at the end of the drought. The crop during the experiment was winter wheat (*Triticum aestivum, RGT Reform*), which was sown on October 28, 2020, and harvested on July 30, 2021.

### 2.3 ^13^C Pulse Labelling; Plant and Soil Sampling

In 2021, plants were pulse labelled at three time points within the vegetation period with ^13^C‐enriched CO_2_ provided by stepwise addition of phosphoric acid (H_3_PO_4_; 85%) to ^13^C‐labelled sodium bicarbonate (NaHCO_3_): (1) before drought (*D1*; 04‐26‐2021); at peak drought (*D2*; 06‐22‐2021); and after rewetting (*RW*; 06‐24‐2021). The CO_2_ concentration of the atmosphere within the labelling chambers was elevated to 800 ppm and kept at this level for 4 h from 8 AM to 12 PM at each of the three labelling events. To avoid carryover effects of the ^13^C label from soil, each labelling was applied at a different spot within the respective sub‐plot. Before drought (*D1*), 99 atom% labelled NaHCO_3_ was used for labelling. At peak drought (*D2*) and after rewetting (*RW*), the label was reduced to 20 atom%. To compare the data between different time points, ^13^C excess content of the first labeling (before drought; *D1*) was divided by 5.

Two plants were sampled 24 h after each labelling (*D1*, *D2*, and *RW*) from the respective labelled area by hand‐cutting 5 cm above the soil surface. Subsequently, soil cores were sampled (12.5 cm diameter, soil depth of 0–20 cm), including the roots of the two harvested plants. Soils were sieved (< 2 mm) and immediately used for measurements of extractable organic carbon content (EOC), and microbial biomass carbon (C_mic_). Roots were collected during the soil sieving process. Gravimetric soil water content was determined, and soils were stored at −20°C.

### 2.4 Aboveground and Belowground Biomass; Root Sugar Content

Plant material was dried (40°C), milled, and C content as well as ^13^C abundance was determined via an elemental analyzer (Euro EA 3000, Euro Vector, Milan, Italy) coupled with a Delta Plus XP mass spectrometer (Thermo Finnigan MAT, Bremen, Germany). Roots were hand washed with H_2_O_deion_, dried at 40°C for 72 h, weighed, and ground with a ball mill. Carbon and ^13^C content were determined via an elemental analyzer (Euro EA 3000, Euro Vector, Milan, Italy) coupled with a Delta Plus XP mass spectrometer (Thermo Finnigan MAT, Bremen, Germany).

Primary root sugars, as well as their ^13^C content, were measured, using them as a proxy for plant‐derived C sources for microbial assimilation and respiration. ^13^C content was determined for sucrose, glucose, and fructose; contents of raffinose and myo‐Inositol were below detection limit. Primary root sugars were extracted and analyzed by a modified method of Richter et al. (2009). In brief, 40 mg of milled root material was extracted with 1 mL of methanol/chloroform/water (12:3:5, v:v:v) for 30 min in a 70°C water bath. After cooling, samples were centrifuged at 10,000 *g* for 2 min and 800 μL of the supernatant was transferred into a new vial. The 800 μL of water and the first 250 μL, and later 500 μL of chloroform, were added for phase separation. In the following, the supernatant was deionized and purified using ion‐exchange cartridges (OnGuard II H cation exchange and OnGuard II anion exchange cartridges; Dionex, Thermo Scientific, Vienna, Austria). Samples were frozen at −20°C. For compound‐specific stable isotope analysis, the unfrozen neutral fractions were analyzed via HPLC‐IRMS as described in detail by Wild et al. (2010).

### 2.5 Soil Respiration

In situ soil respiration measurements were conducted throughout the drought experiment 24 and 72 h after each labeling. Gas samples were taken within each subplot via closed chambers (inner volumes 4850 cm^3^ covered area of 270 cm^2^). Gas samples (20 mL) were taken 0, 10, 20, and 30 min after closure with a syringe via a three‐way stopcock and injected into pre‐evacuated 12 mL exetainers (Labco Ltd., UK). Concentrations of CO_2_ were determined on a gas chromatograph (Agilent Technologies, Santa Clara, USA) equipped with a methanizer and FID for CO_2_. Three external standards were used for calibration by linear regression. ^13^CO_2_ was measured with a mass spectrometer (Thermo Finnigan MAT, Bremen, Germany). The calculation of ^13^CO_2_ was conducted using Equation (1). Here, instead of fumigated and non‐fumigated samples, the ^13^C values of the gas samples taken from each closed chamber 0 and 30 min after closure were used and calculated with the respective CO_2_ concentration.

### 2.6 Microbial Biomass and Extractable Organic Carbon

Microbial biomass carbon content was determined via chloroform‐fumigation‐extraction (CFE) as described by Vance, Brookes, and Jenkinson (1987). Here, 10 g of field fresh soil were fumigated with ethanol free chloroform for 24 h in a desiccator. Samples were then extracted in 40 mL 0.025 M K_2_SO_4_ on a horizontal shaker at 250 rpm for 30 min and centrifuged at 4400 *g* for 30 min. Another 10 g were directly extracted as described above without fumigation and used as non‐fumigated controls and for estimation of extractable organic carbon from soil. The supernatant was filtered at 20 μm. C and N content of the extracts were measured on a total organic carbon analyzer (multi‐N/C 2100S, Analytic Jena AG, Jena, Germany). Microbial biomass C was calculated from the differences in concentration between fumigated and non‐fumigated samples with correction by the *k*_EC_ factor of 0.45 (Joergensen 1996) for C_mic_. For measurement of ^13^C_mic_, 10 mL of the extracts of fumigated and non‐fumigated samples were evaporated at 60°C in a rotary evaporator (RVC 2‐25, Martin Christ, Osterode am Harz, Germany) until only the dry residue remained. The residues were homogenized and weighed into tin capsules with a minimum of 10 μg C per sample. Atom% ^13^C_mic_ was analyzed by elemental analyzer (Euro EA 3000, EuroVector, Milan, Italy) coupled with an isotope ratio mass spectrometer (IRMS, Delta Plus XP, Thermo Finnigan MAT, Bremen, Germany).

The calculation of ^13^C abundance in microbial biomass was done using the following equation:

(1) $$\mathrm{AT}\%C_{\mathrm{mic}}=\frac{\mathrm{AT}{\%}_{f}\times C_{f}-\mathrm{AT}{\%}_{\mathrm{nf}}\times C_{\mathrm{nf}}}{C_{f}-C_{\mathrm{nf}}}$$

where C_f_ and C_nf_ are the extracted organic C content (μg C g^−1^ dry soil) of the fumigated and non‐fumigated samples, and AT%_f_ and AT%_nf_ are the corresponding atom% ^13^C values.

### 2.7 Main Microbial Groups (PLFAs)

Phospholipid fatty acids (PLFAs) were used to quantify the abundance of fungi, gram‐positive, and gram‐negative bacteria and the uptake of recently assimilated C into these groups. PLFAs were assigned according to Kandeler (2015) with gram‐positive bacteria indicated by the PLFAs a15:0, i15:0, i16:0, and i17:0. PLFAs cy17:0 and cy19:0 indicated gram‐negative bacteria. The PLFA 16:1ω5 was included in the calculation of the sum of total microbial PLFAs that occur in both bacteria and fungi (Olsson and Lekberg 2022). The biomarker 18:2ω6.9c was used as a fungal indicator. In short, PLFAs were extracted from 4 g soil with a mixture of chloroform, methanol and citrate buffer (pH = 4) at a ratio of 1:2:0.8 (v/v/v) according to Bligh and Dyer (1959). Following the procedure of Frostegård, Bååth, and Tunlio (1993), the extract was purified using silica gel SPE cartridges (Bond Elut SI, 500 mg, 3 mL, Agilent Technologies, Santa Clara, CA, USA) and separated into glyco‐, neutral, and phospholipid fatty acids. Subsequently, PLFAs were transformed into fatty acid methyl esters (FAMEs) via alkaline methanolysis and quantified by gas chromatography (GC‐FID; Perkin‐Elmer Corporation, Norwalk, CT, USA).

For identification of the fatty acid peaks, samples with a standard qualitative bacterial acid methyl ester mix (BAC mix) and a standard qualitative fatty acid methyl ester mix (FAME mix; both Sigma Aldrich, St. Louis, USA) were used. To achieve baseline separation of peaks during ^13^C analysis of PLFA samples, a four‐step fractionation in Ag^+^‐SPE cartridges (6 mL, Supelco, Palo Alto, USA) was carried out using elution steps with n‐hexane and increasing concentrations of acetone (99:1, v/v; 96:4, v/v; 90:10, v/v; and 0:100, v/v) according to Kramer et al. (2008). Monoenoic trans‐ and cis‐FAMEs (2nd and 3rd fractions) were removed, and only the saturated (first fraction) and dienoic FAMEs (4th fraction) were kept. ^13^C values of FAMEs were determined using a gas chromatograph (6890 series, Agilent Technologies, Santa Clara, CA, USA) coupled to a gas chromatography incinerator III Interphase (Thermo Finnigan, Waltham, MA, USA) connected to a Delta Plus XP mass spectrometer (Thermo Finnigan MAT, Bremen, Germany). ^13^C values of all FAMEs were corrected for the addition of a methyl group according to Denef et al. (2007).

### 2.8 Calculation of Excess ^13^C

The excess ^13^C, i.e., the labeling‐derived amount of ^13^C in individual C pool stocks (mg ^13^C m^−2^) was calculated as follows:
(2)

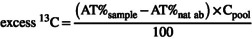

where AT%_sample_ is the atom% of the labelled sample and AT%_nat ab_ is the atom% of the respective corresponding sample from the same plot without labelling. C_pool_ is the respective C‐pool (plant, root sugars, C_mic_, EOC, PLFAs, CO_2_). C‐pools were calculated per m^2^ with a soil depth of 20 cm and bulk density of 1.21 g cm^−3^.

### 2.9 Statistics

Data were analyzed using a mixed model approach. The design was a split plot with a main plot factor temperature (*T*_*i*_ with levels *Ta*: ambient temperature and *Te*: elevated temperature) and a subplot factor precipitation (*P*_*j*_ with levels *Pa*: ambient precipitation and *Pr*: reduced precipitation). As data were taken repeatedly over time, the day of measurement (*D*_*k*_ with three levels: *D1*, *D2*, and *RW*) was the repeated measures factor, and correlations between corresponding random and error effects of all time points were assumed. The model can be described as follows:

$$\begin{array}{c} y_{\text{hijk}}=\mu +b_{h}+T_{i}+P_{j}+D_{k}+{\left(\mathrm{TD}\right)}_{\mathrm{ik}}\\ \;+{\left(\mathrm{PD}\right)}_{\mathrm{jk}}+{\left(\mathrm{TP}\right)}_{\mathrm{ij}}+{\left(\mathrm{TPD}\right)}_{\mathrm{ijk}}+p_{\mathrm{hi}}+e_{\text{hijk}} \end{array}$$

where $y_{\text{hijk}}$ is the observation of the *i*th temperature, *j*th precipitation level of the *h*th block at day *k*, μ is the intercept, and $b_{h}$ is the random effect of the *h*th complete block. $T_{i}$, $P_{j}$, and $D_{k}$ are the fixed main effects for the *i*th temperature, *j*th precipitation level, and *k*th day, respectively. ${\left(\mathrm{TD}\right)}_{\mathrm{ik}}$, ${\left(\mathrm{PD}\right)}_{\mathrm{jk}}$, ${\left(\mathrm{TP}\right)}_{\mathrm{ij}}$, ${\left(\mathrm{TPD}\right)}_{\mathrm{ijk}}$ are the fixed two‐ and three‐way interactions of the corresponding factors involved, and $p_{\mathrm{hi}}$ and $e_{\text{hijk}}$ are the respective main‐ and sub‐plot errors. To account for potential correlation of errors, a first‐order autoregressive variance–covariance matrix with both homogeneous and heterogeneous day‐specific variances was fitted. The best‐fitting model was selected via AIC (Wolfinger 1993). To account for the assumptions of the mixed model analysis, residuals were checked graphically for normal distribution and deviations from variance assumptions made within the model (Kozak, Nagel, and Santosh 2018). Afterwards, the model was reduced by dropping non‐significant fixed effects, starting with the highest‐level interaction term (${\left(\mathrm{TPD}\right)}_{\mathrm{ijk}}$). Backward selection with the *p*‐values of the corresponding *F* tests was performed until all effects remaining in the model were significant. Note that main effects and lower‐level interactions were not considered if a higher‐level interaction existed in the model. Further note that block effects of block, main plot, and error were not selected and thus remained in the final model. Data was analyzed using the package ‘asreml’ (version 4.1.0.189) for R (version 3.6.3 (R Core Team 2023)).

## 3 Results

### 3.1 Soil Moisture and Temperature Conditions

**FIGURE 1:**
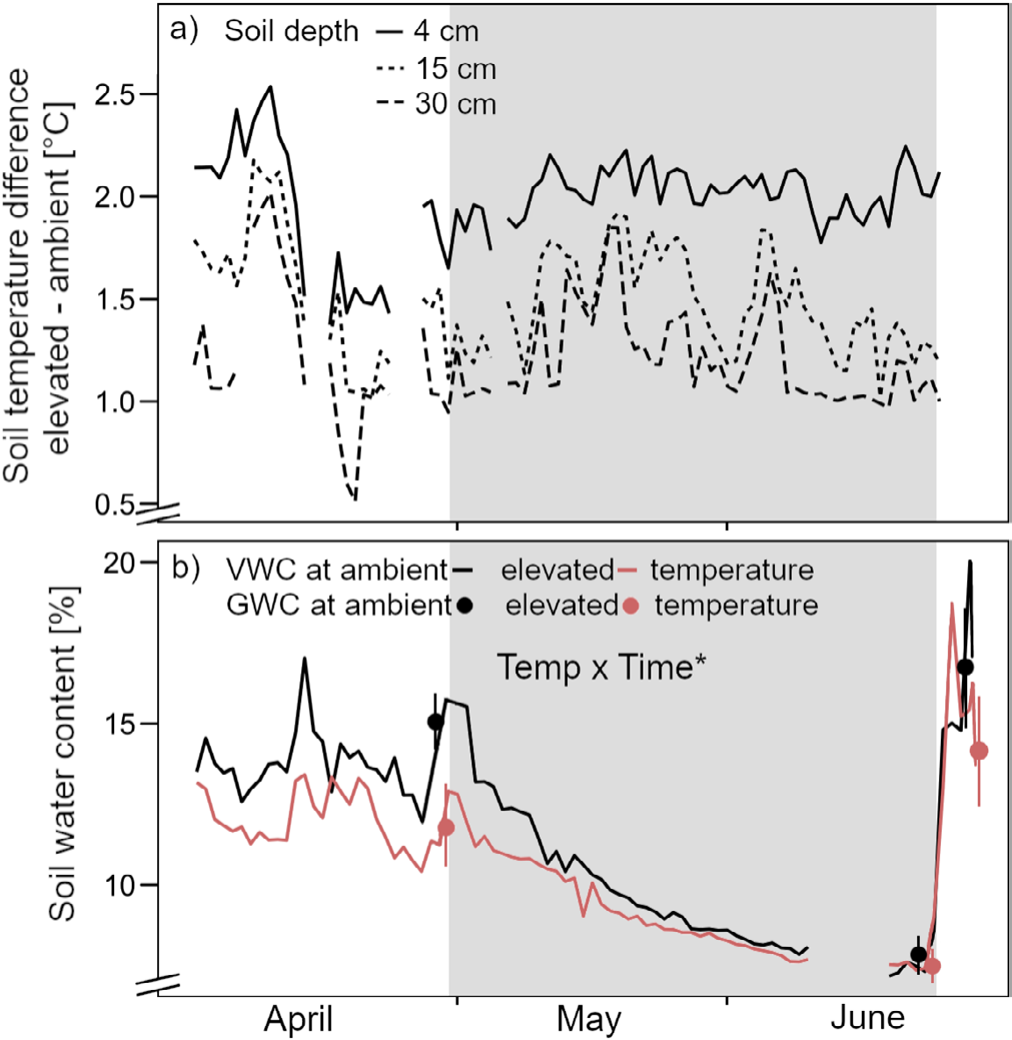
(a) Differences in soil temperature between warmed and ambient temperature soils at soil depths of 4, 15, and 30 cm. (b) Volumetric water content (VWC; line; 0–15 cm) and gravimetric water content (GWC; dot; 0–20 cm) at ambient (black) and elevated (red) temperatures. Gravimetric water content was determined at different sampling dates (D1: Before drought; 04‐26‐2021, D2: Peak drought; 06‐22‐2021, RW: After rewetting; 06‐24‐2021) with data presented as mean values with standard errors (*n* = 8). The grey background marks the drought period. Declarations on statistics refer to model output across all time points within each parameter (*p* < 0.05*).

The temperature difference between warmed and ambient temperature soils was on average 2°C at 4 cm soil depth, 1.5°C at 15 cm depth, and 1.1°C at 30 cm depth (Figure 1a). In the weeks before drought, volumetric soil water content (VWC) was on average 14% in ambient temperature soils and 12% in warmed soils (Figure 1b). As drought progressed, the difference between ambient temperature and warmed soils diminished, and soils reached a minimum of 5% VWC at peak drought conditions. Following rewetting, VWC spiked to its highest level of 19%. The dynamic in VWC was also reflected in the gravimetric water content of the soil samples with significantly lower values in warmed soils at the beginning of the drought and after rewetting (Figure 1b).

### 3.2 Plant Performance

**FIGURE 2:**
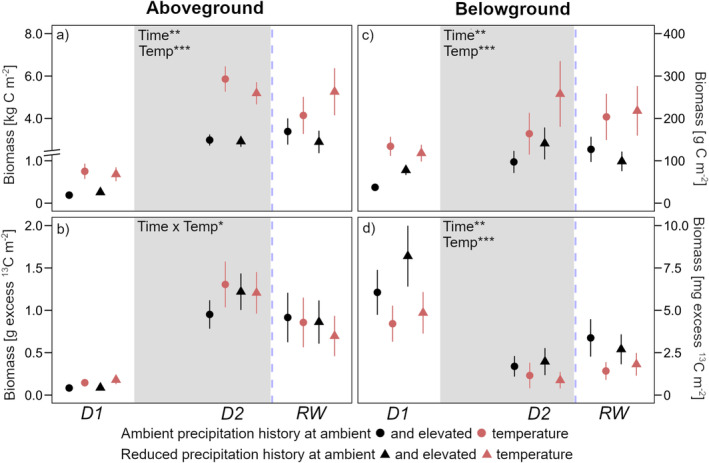
(a) Aboveground plant biomass; (b) with excess ^13^C in aboveground plant biomass; (c) belowground plant biomass; (d) with excess ^13^C in belowground plant biomass. Sampling dates were D1: Before drought, D2: Peak drought, RW: After rewetting. Precipitation history (ambient; reduced) at ambient (black) and elevated (red) temperatures are the long‐term treatments. The grey background marks the drought period. Data are presented as mean values with standard errors (*n* = 4). Declarations on statistics refer to model output across all time points within each parameter (*p* < 0.05*; *p* < 0.01**; *p* < 0.001***).

Over time, aboveground biomass increased about 10‐fold while belowground biomass about doubled as peak drought was reached (Figure 2a,c). At all timepoints, warming increased both above‐ and belowground biomass. ^13^C allocation to aboveground biomass increased in proportion to the total aboveground biomass (Figure 2b). ^13^C allocation to root biomass was strongly reduced at peak drought compared to pre‐drought and remained at low levels after rewetting (Figure 2d). At all timepoints, warming decreased excess ^13^C in root biomass.

**FIGURE 3:**
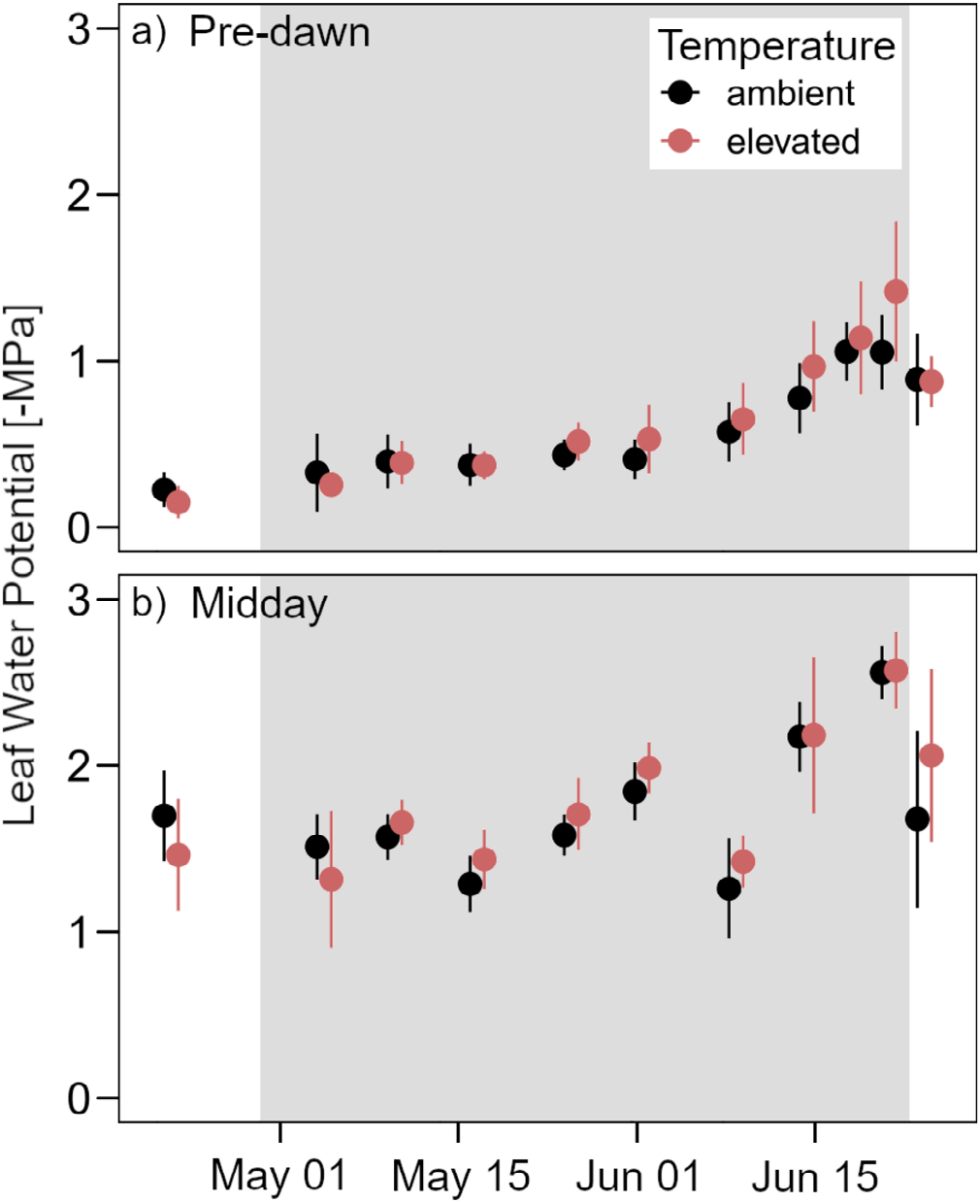
Leaf water potential from ambient temperature (black) and warmed (red) plots at (a) pre‐dawn and (b) midday. The grey background marks the drought period. Data are presented as mean values with standard errors (*n* = 8).

Pre‐dawn and midday leaf water potential peaked at −1.2 and −2.5 MPa, respectively (Figure 3a,b). Overall, although warming decreased soil moisture content throughout the drought experiment, warming did not affect leaf water potential. Historical precipitation treatments also had no effect on leaf water potential.

### 3.3 Root Sugars

**FIGURE 4:**
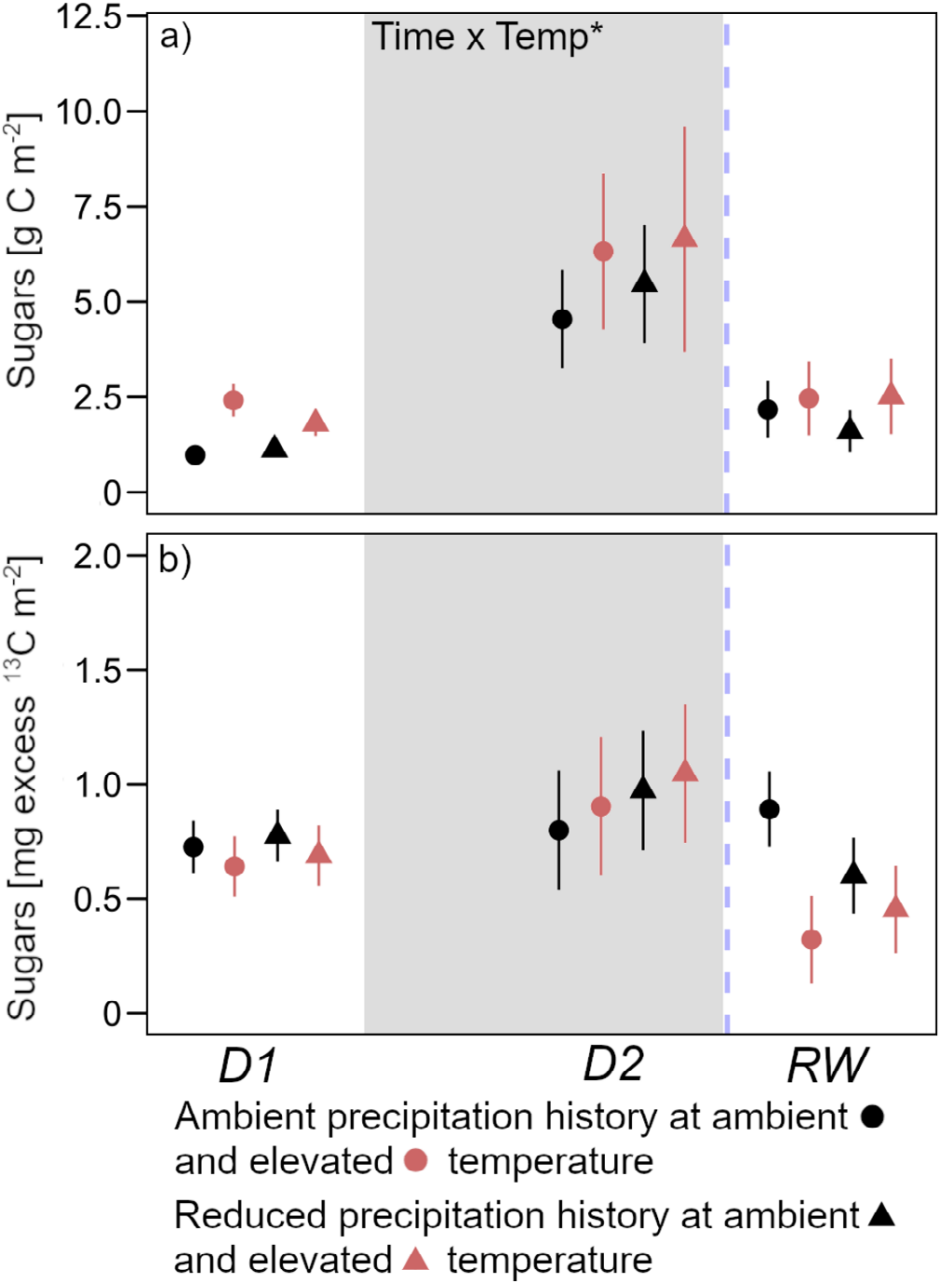
(a) Root sugar content with (b) excess ^13^C in root sugars. Sampling dates were D1: Before drought, D2: Peak drought, RW: After rewetting, at a soil depth of 0–20 cm. Precipitation history (ambient; reduced) at ambient (black) and elevated (red) temperatures are the long‐term treatments. The grey background marks the drought period. Data are presented as mean values with standard errors (*n* = 4). Declarations on statistics refer to model output across all time points (*p* < 0.05*).

With plant growth, total root sugar content increased three‐ to fourfold by peak drought and decreased to around pre‐drought levels in response to rewetting (Figure 4a). At all three time points, warming increased sugar content; however, this increase was only significant (+110% on average) before drought. At peak drought, ^13^C allocation to sugars remained similar to its level under pre‐drought conditions (Figure 4b). After rewetting, ^13^C allocated to sugars remained at similar levels in ambient temperature soils and decreased in warmed soils compared to peak drought conditions.

### 3.4 Total and Extractable Organic Carbon; Microbial Biomass Carbon

**FIGURE 5:**
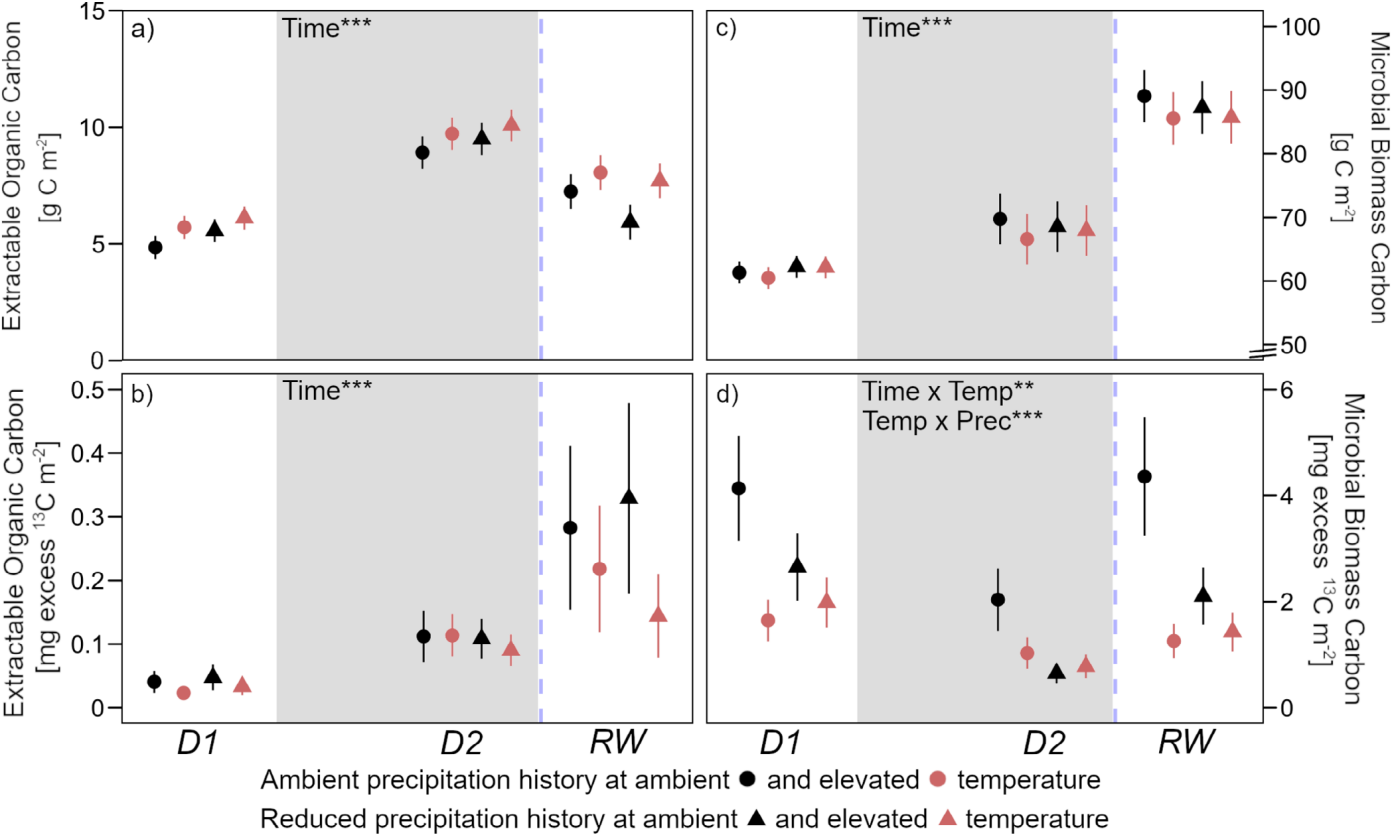
(a) Extractable organic carbon (EOC) with (b) excess ^13^C in EOC; (c) Microbial biomass carbon (C_mic_) with (d) excess ^13^C in C_mic_. Sampling dates were D1: Before drought, D2: Peak drought, RW: After rewetting, at a soil depth of 0–20 cm. Precipitation history (ambient; reduced) at ambient (black) and elevated (red) temperature are the long‐term treatments. The grey background marks the drought period. Data are presented as mean values with standard errors (*n* = 4). Declarations on statistics refer to model output across all time points within each parameter (*p* < 0.01**; *p* < 0.001***).

EOC content increased as peak drought was reached (+80% on average; Figure 5a) and decreased in response to rewetting by 20%–40% compared to peak drought levels. Warming tended overall (*p* = 0.1) to increase EOC content by 10%–50%. ^13^C allocation to EOC doubled to quadrupled from beginning of drought to peak drought (*D1* to *D2*), and further from peak drought to rewetting (*D2* to *RW*; Figure 5b). Warming tended (*p* = 0.1) to decrease excess ^13^C in the EOC pool at all time points (*D1*: −40%; *D2*: −27%; *RW*: −15% to −53%).

C_mic_ content increased by 10%–25% by peak drought (Figure 5c). Rewetting induced a further significant increase in C_mic_ content of 25%–40%. Neither warming nor precipitation history had an effect on C_mic_ content over the course of the experiment. ^13^C allocation to C_mic_ decreased as drought progressed (−35% to −75%) but returned to pre‐drought levels in response to rewetting (Figure 5d). On almost every sampling occasion, warming decreased ^13^C allocation to C_mic_. While this was only a tendency (*p* = 0.1) in soils with reduced precipitation history, the effect of warming was considerably larger and significant (around −60%) in soils with ambient precipitation history. Altogether, excess ^13^C in C_mic_ was at all time points significantly highest in the long‐term wettest soils (ambient precipitation history at ambient temperature).

### 3.5 PLFAs

**FIGURE 6:**
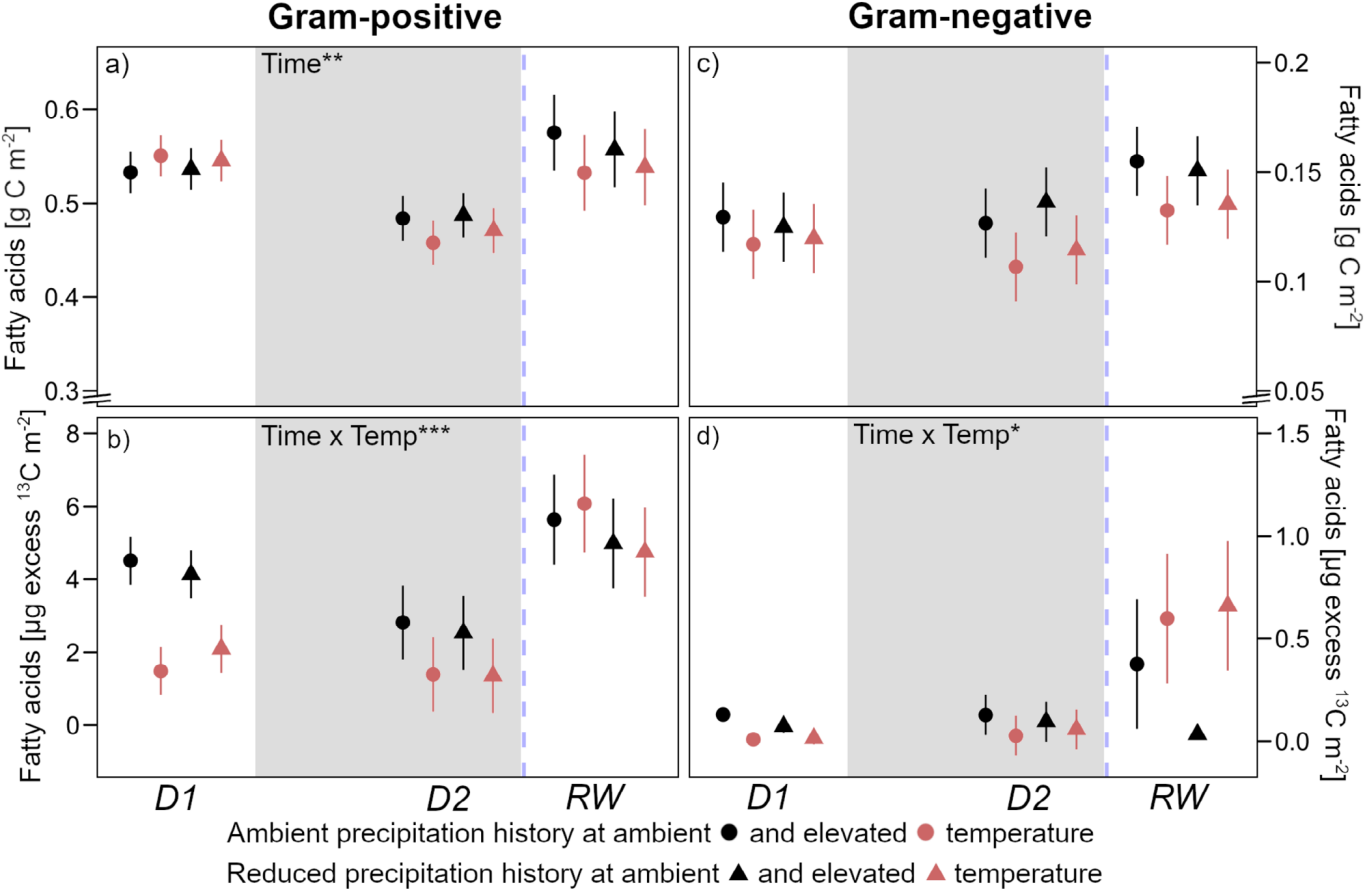
(a) Amount of C in fatty acids indicative of gram‐positive bacteria with (b) excess ^13^C in these fatty acids; (c) amount of C in fatty acids indicative of gram‐negative bacteria with (d) excess ^13^C in these fatty acids. Sampling dates were D1: Before drought, D2: Peak drought, RW: After rewetting, at a soil depth of 0–20 cm. Precipitation history (ambient; reduced) at ambient (black) and elevated (red) temperature are the long‐term treatments. The grey background marks the drought period. Data are presented as mean values with standard errors (*n* = 4). Declarations on statistics refer to model output across all time points within each parameter (*p* < 0.05*; *p* < 0.01**; *p* < 0.001***).

Drought significantly reduced gram‐positive bacteria by 10%–15% but not gram‐negative bacteria (Figure 6a,c). In response to rewetting, overall bacterial levels increased by around 20%. Except for gram‐positive bacteria before drought, warming tended (*p* = 0.1) to decrease bacterial levels at every timepoint. As peak drought was reached, ^13^C allocated in both bacterial groups was either similar (gram‐negative) or tended (*p* = 0.1) to decrease (Figure 6b,d). In response to rewetting, ^13^C allocation to both bacterial groups significantly increased ten‐ to twentyfold compared to peak drought in most soils.

**FIGURE 7:**
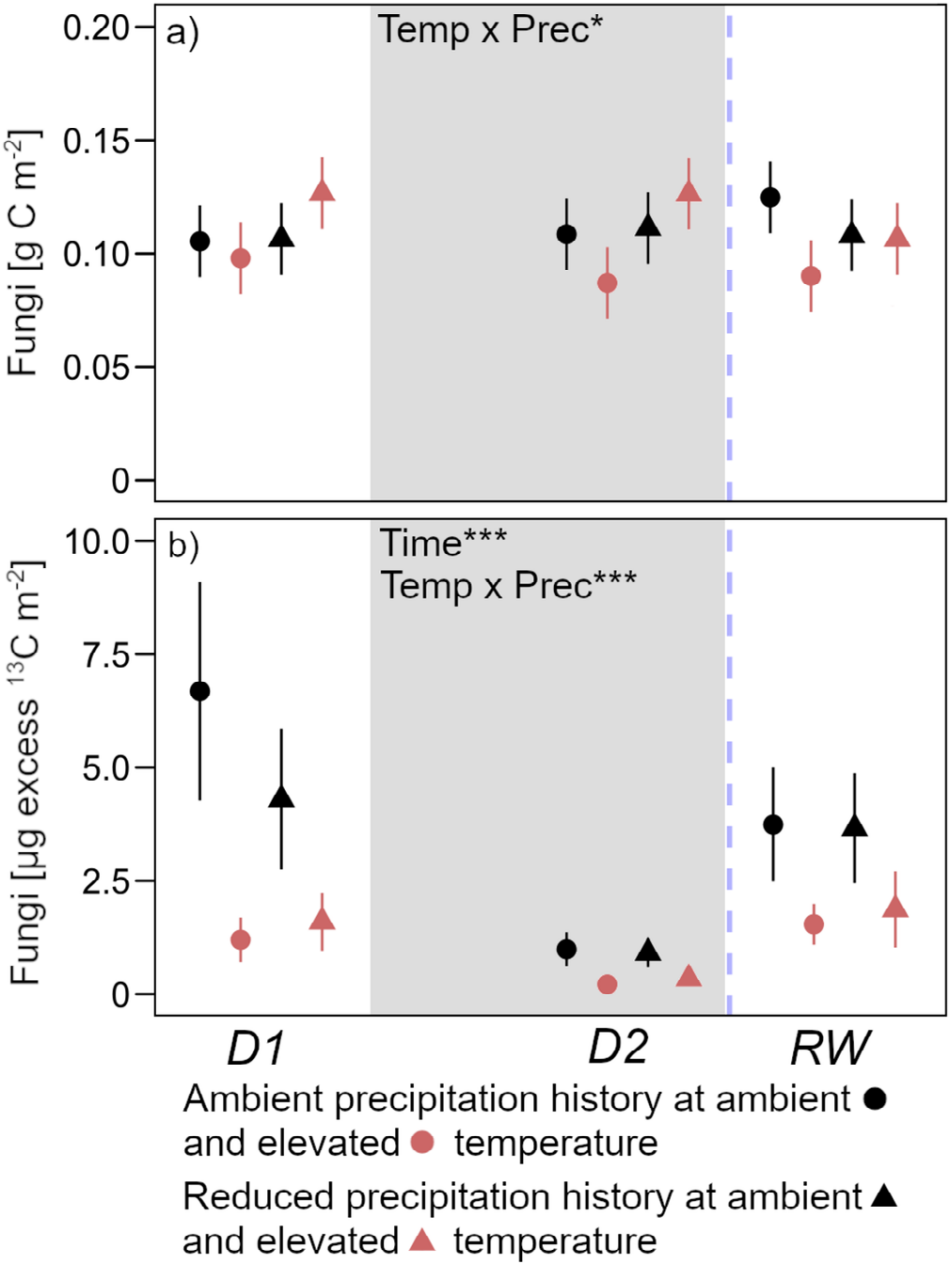
(a) Amount of C in the fungal fatty acid 18:2ω6,9 with (b) excess ^13^C in this fatty acid. Sampling dates were D1: Before drought, D2: Peak drought, RW: After rewetting, at a soil depth of 0–20 cm. Precipitation history (ambient; reduced) at ambient (black) and elevated (red) temperature are the long‐term treatments. The grey background marks the drought period. Data are presented as mean values with standard errors (*n* = 4). Declarations on statistics refer to model output across all time points (*p* < 0.05*; *p* < 0.001***).

Fungal biomass largely remained stable over the course of the experiment (Figure 7a). Warming reduced fungal biomass in soils with ambient precipitation history, however, exhibiting the opposite effect in soils with a history of reduced precipitation. ^13^C allocation to fungal biomass decreased as peak drought was reached (−85%) but returned to around pre‐drought levels in response to rewetting (Figure 7). Furthermore, warming significantly reduced excess ^13^C in fungal biomass by, on average, 70% at each timepoint.

### 3.6 Soil Respiration

**FIGURE 8:**
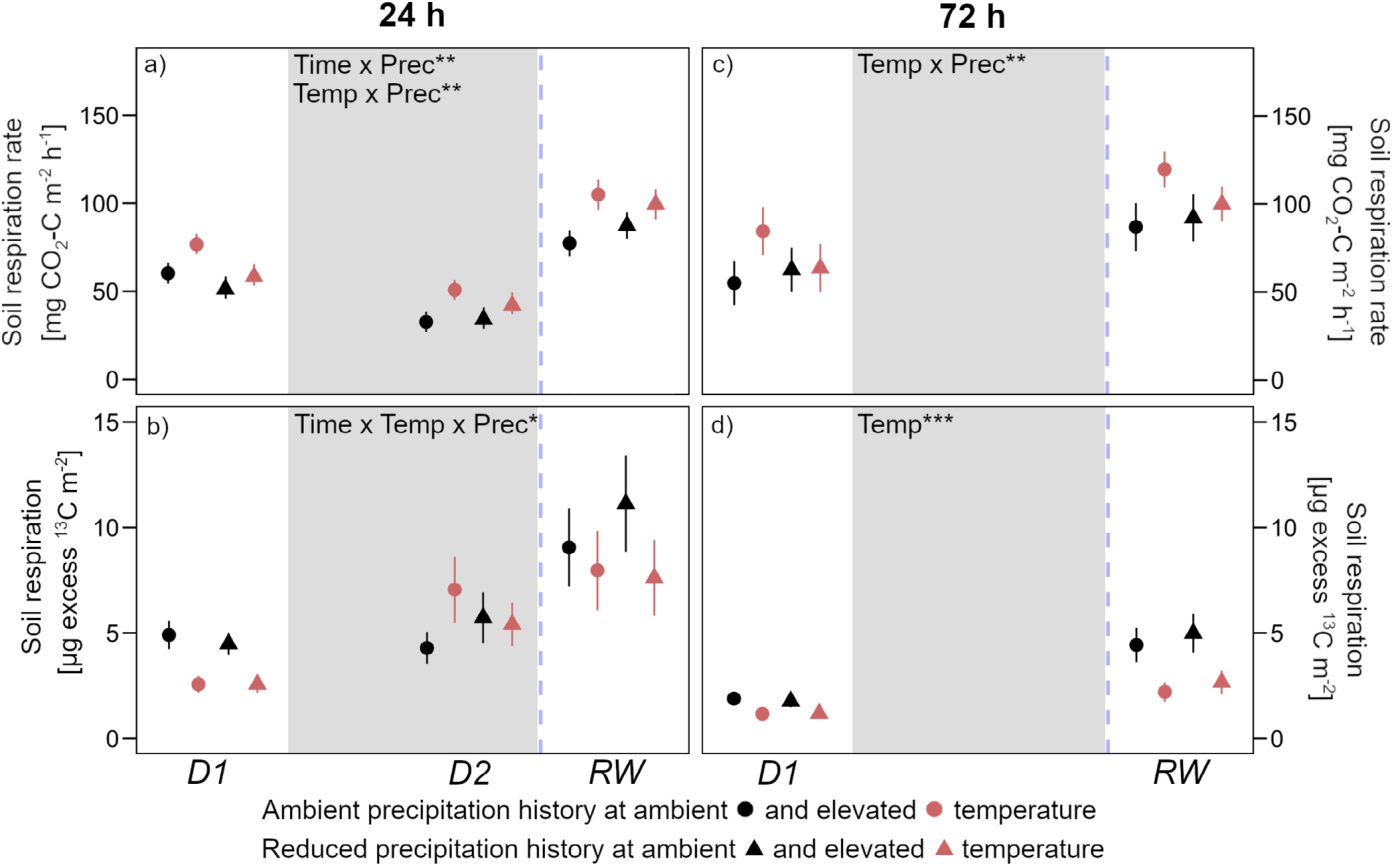
Soil respiration (a) 24 h and (c) 72 h after pulse labeling. Excess ^13^C in soil respiration (b) 24 h and (d) 3 days after pulse labeling. Sampling dates were D1: Before drought, D2: Peak drought, RW: After rewetting. Precipitation history (ambient; reduced) at ambient (black) and elevated (red) temperatures are the long‐term treatments. Grey background marks the drought period. Data are presented as mean values with standard errors (*n* = 4). Declarations on statistics refer to model output across all timepoints within each parameter (*p* < 0.05*; *p* < 0.01**; *p* < 0.001***).

Soil respiration decreased in response to drought (−35% on average) and subsequently more than doubled following rewetting in relation to peak drought levels (Figure 8a). Compared to pre‐drought respiration, this was an increase of 30% in ambient temperature and 70% in warmed soils, and this increase was still present 72 h after rewetting (Figure 8c). At every timepoint, warming increased respiration. However, this was only significant in soils with ambient precipitation history, while in those with reduced precipitation history, the warming effect was less pronounced (Figure 8a,b). Overall, ^13^C allocation to respiration increased from pre‐drought to peak drought (Figure 8b). When soils were rewetted, ^13^C allocation to respiration remained at peak drought levels in ambient temperature soils but decreased in warmed soils. Warming reduced excess ^13^C when soils were relatively wet during the experiment (*D1* and *RW*), but not in dry soils at D2 (Figure 8b). At *D2* after 72 h, no measurement was possible as these soils were already rewetted at this timepoint.

## 4 Discussion

### 4.1 Belowground C Allocation

Six months after sowing, plant biomass was substantially higher in warmed soils at the beginning of the drought experiment, although the 24‐h snapshot of pulse labelling showed only small differences in ^13^C uptake between warmed and ambient temperature soils (Figure 2a,b). In the temperate agroecosystem, the winter months are sufficiently wet and, therefore, soil temperature can be considered as the limiting factor for crop growth in the early stages of development (Gavito et al. 2001). Over the course of the experiment, total root biomass increased, and so did root sugar concentration (Figures 2c and 4a). However, at peak drought, when plants were severely drought stressed, overall C allocation to roots substantially decreased (Figure 2d) even though plants continuously assimilated C in proportion to their aboveground biomass growth (Figure 2b). This decrease can on the one hand, be attributed to a shift in ^13^C allocation towards the ear, the organ that is the main photosynthetic contributor to grain filling during drought (Li et al. 2017). On the other hand, reduced phloem transport during drought (Sevanto 2018) may have hampered the translocation of photo‐assimilates to root biomass. Besides, root growth of winter wheat can reach a soil depth well beyond the 20 cm we sampled (Thorup‐Kristensen, Salmerón Cortasa, and Loges 2009); ^13^C could therefore have been allocated to deeper root zones. However, the previous study also showed that the majority of winter wheat root biomass is found in the upper 20 cm, which is consistent with results showing a strong decrease (−90%) in wheat root biomass below 15 cm soil depth (Giongo et al. 2024).

Although overall significantly less ^13^C was translocated to the roots during peak drought compared to before drought, ^13^C in the root sugar pool tended to increase in some soils, which was also reflected in the ^13^C content of the EOC pool in soil (Figures 4b and 5b). This may have been due to the fact that under drought stress, plants allocate a higher proportion of recently assimilated C to exudates as opposed to root structural development (Karst et al. 2017; Preece et al. 2018). The overall concomitant increases in the sugar and EOC pools during drought as well as their subsequent decrease after rewetting (Figures 4a and 5a) are in accordance with previous studies (Deng et al. 2021; Evans, Allison, and Hawkes 2022). It indicates that the exudation of sugars constitutes a major pathway of belowground C input and is important for labile C dynamics in arable soils with low SOC content (Villarino et al. 2021). In more detail, the increase in ^13^C in the EOC pool as peak drought approached was much larger than in the root sugar pool (Figures 4b and 5b). This accumulation of ^13^C in EOC could be explained by a decoupling of root exudation and microbial EOC utilization, which was also linked to a decrease in respiration at peak drought (Figure 8a). The following rewetting led to immediate resuscitation of microbial activity, as microbes made quick use of the labile C which had accumulated during the drought (Schimel, Balser, and Wallenstein 2007; Schimel 2018). However, it is interesting to note that ^13^C allocated to the labile C pool significantly increased in response to rewetting while at the same time it decreased in root sugars (Figures 4b and 5b). This suggests that 24 h after rewetting, the ^13^C pulse had already passed the roots and appeared in the labile C pool (Karlowsky et al. 2018).

### 4.2 Microbial C Utilization

The carbon translocated to belowground can be used by soil microorganisms for the synthesis of new microbial cell walls (PLFAs) during growth (Kaiser et al. 2015), or for osmotic adjustment via intracellular C accumulation in the cytoplasm. Our findings of increasing intracellular C (C_mic_; Figure 5c) at a simultaneously stable or decreasing PLFA‐C pool size at peak drought (Figures 6a,c and 7a) may, therefore, be attributed to microbial accumulation of osmolyte compounds as a stress response to decreasing water potential (Schimel 2018). Although not strong, but significant, we were surprised to find that drought had reduced the abundance of gram‐positive bacteria, despite their theoretically higher drought resistance due to thick cell membranes (Hartmann et al. 2017). Still, gram‐positive bacteria have shown a decrease in growth during drought (Canarini et al. 2024) which may explain their decrease in abundance, however so have gram‐negative bacteria which remained unaffected in abundance during drought in our experiment. Although it is difficult to find a biological reason for our observation, there have been findings where, in line with our results, drought decreased the gram‐positive to gram‐negative bacterial ratio (Preece et al. 2019). The fact that C_mic_ and bacterial PLFA levels increased uniformly shortly after rewetting indicated that internal C allocation was rearranged to support growth again; this fits with previously observed recovery of bacterial growth 24 h after rewetting (Göransson et al. 2013; Meisner et al. 2017).

We hypothesized that microbial communities with a history of reduced summer precipitation would be more efficient in assimilating C during extreme drought; i.e., they would maintain higher microbial biomass compared to microbes in long‐term wetter soils. However, this was not the case. Neither the response of microbial biomass nor of main microbial groups during the drought differed between soils exposed to ambient or altered mean precipitation (Figures 5a, 6a,b, and 7a). Interestingly, we found that fungal biomass in warmed soils was significantly higher when soils had been exposed to reduced mean summer precipitation (Figure 7a). This contrasts with previous studies that found decreasing fungal abundance under long‐term warming (Frey et al. 2008; Wu et al. 2022). In our experiment, reduced summer precipitation modulated the effect of warming but could not, however, be explained by either a difference in the amount of recently assimilated C (Figure 7b) or overall substrate availability (Figure 5a). In addition, a significant shift in fungal communities following repeated moderate precipitation reduction is very unlikely (Albracht et al. 2023). We can therefore only speculate that net drier conditions via the combination of reduced summer precipitation and enhanced desiccation following warming overcame a threshold and led to increased fungal abundance in these soils.

Aside from this, precipitation history did not affect the overall microbial C pool dynamics throughout the experiment. However, we found indications of a legacy effect driving C assimilation by microbes. In detail, a history of reduced summer precipitation significantly reduced the allocation of recently assimilated C to the microbial cytoplasm (Figure 5d). In a recent study, a repeated precipitation reduction during the growing season increased the relative abundance of oligotrophic, slow‐growing bacterial phyla (Li et al. 2022). These phyla exhibit relatively low dependency on plant‐derived C and we therefore speculate that in our field, drier summer months favored such bacterial phyla, e.g., *Acidobacteria* (Castro et al. 2010).

In addition to the above‐mentioned mechanisms with respect to precipitation history, our data suggest warming induces profound changes in how microbes use and allocate C. First, microbial communities in warmed and ambient temperature soils built similar total biomass (Figure 5c); however, there was slightly greater substrate availability in warmed soils throughout the experiment (Figure 5a). This accords with what has previously been found in this field (Leyrer et al. 2022) and—together with our respiration data—suggests overall lower microbial efficiency at building biomass in warmed soils. Second, although the total C pool sizes of microbial biomass did not differ between soils, bacteria and fungi in warmed soils used less plant‐derived C to build biomass than in ambient temperature soils, even though these compounds were available as indicated by the EOC pool (Figure 5b). One possible explanation is that microbes in warmed soils grow more slowly (Liu et al. 2024), reducing incorporation of the ^13^C signal into the total microbial biomass pool during a short‐term pulse labeling. This would, however, contrast findings of experiments showing that decadal warming increases microbial growth rates (Walker et al. 2018). If growth characteristics do not explain the differences between warmed and ambient temperature soils, warming could have shifted microbial C acquisition toward older C sources from the SOC pool. This has indeed been shown previously, especially for fungi (Streit et al. 2014). Additionally, in agreement with our previous discussion on the legacy effects of reduced summer precipitation, decadal warming of a temperate forest also shifted the microbial community toward bacteria favoring oligotrophic growth strategies that do not rely on easily usable substrates like root sugars (DeAngelis et al. 2015).

In terms of the observed respiration dynamics, our data showed an expected pattern, with a decrease approaching peak drought and the characteristic ‘Birch effect’ (Birch 1958) following rewetting (Figure 8a). This respiration pulse was, in contrast to our assumption, independent of the long‐term mean climatic conditions in which long‐term wetter soils had no stronger respiration response to rewetting after drought than did drier soils (Figure 8a). However, as our first measurement of soil respiration was conducted 24 h after rewetting, we probably did not capture the immediate respiration response. As a consequence, more distinct differences between soils with varying precipitation histories cannot be excluded (Meisner, Rousk, and Bååth 2015). Nevertheless, corresponding with microbial biomass, we found the legacy of reduced summer precipitation to affect the soil respiration dynamics in our experiment. Although warming, as also shown in many studies (Maes et al. 2024), increased respiration, the legacy of reduced summer precipitation inhibited the stimulating effect of warming (Figure 8a,c). This fits with our observed highest fungal biomass in the long‐term driest soils, since fungi have been shown to use C efficiently for growth rather than for energy metabolism (Malik et al. 2016). In addition, the respiration data were broadly in line with the C allocation dynamics observed in the microbial biomass: the proportion of recently assimilated C was lower in the respired CO_2_ from warmed soils compared to ambient temperature soils (Figure 8b,d). At samplings before drought and after rewetting, this can be explained by the lower excess ^13^C in the labile C pools (sugars, EOC; Figures 4a,b and 5a,b). Consistent with this is the contrasting pattern at peak drought, where differences in respiration are largely represented by differences in ^13^C allocation to respiration. This connects well to the fact that at peak drought, the ^13^C label of the sugars paralleled the root sugar pool.

We conclude that in a future overall warmer and drier world, plant–soil C allocation during extreme drought will differ from what we observe today. We base this on our findings where over a decade of drier summer months via reduced precipitation imposed legacy effects on microbial C allocation dynamics during drought. These effects were, in some cases, modulated by soil warming, the most important factor in our experiment. Temperature elevation generally increased the soil C inputs (root biomass, rhizodeposits, EOC), as well as output (respiration). We also found indications that warming shifted soil microorganisms toward use of SOC, rather than plant‐derived C, to respire and build biomass during drought. These findings highlight that anticipating the impact of future extreme drought on C cycling dynamics within plant–soil systems requires integration of the soil's precipitation history, the expected concurrent temperature increase, and their interaction.

## Author Contributions

**Vinzent Leyrer:** data curation, formal analysis, funding acquisition, investigation, project administration, visualization, writing – original draft, writing – review and editing. **Juliette Blum:** investigation, project administration, writing – review and editing. **Sven Marhan:** conceptualization, project administration, supervision, writing – review and editing. **Ellen Kandeler:** project administration, resources, supervision, writing – review and editing. **Telse Zimmermann:** investigation, writing – review and editing. **Bernd J. Berauer:** investigation, writing – review and editing. **Andreas H. Schweiger:** conceptualization, writing – review and editing. **Alberto Canarini:** supervision, writing – review and editing. **Andreas Richter:** conceptualization, resources, writing – review and editing. **Christian Poll:** conceptualization, funding acquisition, project administration, supervision, writing – review and editing.

## Conflicts of Interest

The authors declare no conflicts of interest.

## Supporting information


Table S1.

## Data Availability

The data that support the findings of this study and R code used to analyze the data are openly available in Zenodo at https://doi.org/10.5281/zenodo.14730497.
