# Supplementary material for: Drought Impacts on Plant–Soil Carbon Allocation—Integrating Future Mean Climatic Conditions
Source: Glob Chang Biol. 2025 Feb 12;31(2):e70070. doi: 10.1111/gcb.70070 (PMC11815358; doi:10.1111/gcb.70070)
Supplement: Supplementary file 1 — Table S1. [file GCB-31-e70070-s001.docx]

Tab. S 1: Original data of the measured parameters in the drought experiment.

| Plot No. | Temperature |  | Precipitation | Timepoint | Plant BM [g DW plant^-1^] | Root BM [g DW plant^-1^] | Sugars [mg l^-1^] | Cmic [µg g^-1^ DW] | EOC [µg g^-1^ DW] | Respiration [mmol CO_2_-C m^-2^ h^-1^] | Gram^+^ Bacteria [nmol g^-1^ DW] | Gram^-^ Bacteria [nmol g^-1^ DW] | Fungi [nmol g^-1^ DW] |
| --- | --- | --- | --- | --- | --- | --- | --- | --- | --- | --- | --- | --- | --- |
| 3 | elevated |  | reduced | pre-drought | 3.26 | 0.98 | 86.06 | 284.36 | 32.26 | 62.84 | 14.82 | 2.79 | 3.21 |
| 4 | elevated |  | ambient | pre-drought | 3.18 | 0.85 | 120.69 | 249.38 | 26.02 | 79.10 | 13.05 | 2.38 | 2.16 |
| 9 | ambient |  | ambient | pre-drought | 0.78 | 0.39 | 93.47 | 240.40 | 18.20 | 62.21 | 12.92 | 2.59 | 1.60 |
| 11 | ambient |  | reduced | pre-drought | 1.32 | 0.55 | 77.44 | 260.42 | 21.41 | 56.59 | 12.20 | 2.44 | 2.09 |
| 17 | elevated |  | ambient | pre-drought | 3.91 | 0.57 | 118.02 | 247.32 | 23.60 | 72.74 | 12.64 | 2.37 | 1.90 |
| 18 | elevated |  | reduced | pre-drought | 2.56 | 0.52 | 111.25 | 242.59 | 25.01 | 65.63 | 11.13 | 2.19 | 1.93 |
| 21 | ambient |  | ambient | pre-drought | 0.94 | 0.45 | 107.54 | 238.65 | 18.20 | 60.98 | 11.12 | 2.96 | 2.59 |
| 23 | ambient |  | reduced | pre-drought | 1.32 | 0.46 | 49.56 | 248.43 | 17.84 | 57.12 | 11.65 | 2.34 | 1.75 |
| 28 | elevated |  | ambient | pre-drought | 0.50 | 0.60 | 30.38 | 216.71 | 17.55 | NA | 12.58 | 2.45 | 1.57 |
| 30 | elevated |  | reduced | pre-drought | 1.45 | 0.24 | 85.53 | 268.25 | 22.18 | NA | 12.09 | 2.49 | 2.38 |
| 34 | ambient |  | reduced | pre-drought | 2.22 | 0.56 | 108.64 | 258.56 | 29.09 | 47.19 | 12.48 | 2.58 | 1.84 |
| 36 | ambient |  | ambient | pre-drought | 0.94 | 0.21 | 92.36 | 234.32 | 19.76 | 63.05 | 12.34 | 2.45 | 2.29 |
| 40 | elevated |  | ambient | pre-drought | 2.78 | 0.68 | 106.75 | 241.64 | 19.81 | 78.19 | 11.54 | 2.24 | 1.92 |
| 42 | elevated |  | reduced | pre-drought | 2.64 | 0.67 | 112.15 | 240.73 | 22.40 | 46.44 | 11.26 | 2.19 | 2.24 |
| 45 | ambient |  | reduced | pre-drought | 0.84 | 0.48 | 120.17 | 269.68 | 24.43 | 43.94 | 12.20 | 2.71 | 2.52 |
| 46 | ambient |  | ambient | pre-drought | 1.75 | 0.36 | 111.27 | 255.75 | 24.66 | 54.92 | 11.85 | 2.47 | 1.64 |
| 3 | elevated |  | reduced | peak drought | 21.93 | 1.36 | 417.90 | 336.77 | 65.55 | 34.79 | 11.38 | 2.38 | 2.25 |
| 4 | elevated |  | ambient | peak drought | 25.98 | 0.95 | 383.86 | 244.95 | 37.55 | 58.30 | 11.50 | 2.12 | 1.72 |
| 9 | ambient |  | ambient | peak drought | 15.14 | 1.02 | 310.71 | 299.11 | 34.20 | 34.40 | 11.28 | 2.40 | 1.99 |
| 11 | ambient |  | reduced | peak drought | 16.25 | 0.82 | 365.33 | 302.97 | 46.93 | 40.75 | 10.72 | 2.37 | 2.32 |
| 17 | elevated |  | ambient | peak drought | 19.82 | 0.77 | 279.30 | 246.85 | 50.58 | 47.12 | 10.19 | 1.87 | 1.75 |
| 18 | elevated |  | reduced | peak drought | 18.45 | 1.39 | 182.34 | 269.01 | 65.89 | 49.10 | 10.71 | 2.16 | 2.96 |
| 21 | ambient |  | ambient | peak drought | 12.35 | 0.57 | 330.84 | 261.07 | 41.03 | 34.47 | 9.55 | 2.06 | 1.93 |
| 23 | ambient |  | reduced | peak drought | 15.92 | 1.11 | 355.85 | 254.73 | 36.35 | 40.98 | 9.86 | 3.04 | 2.24 |
| 28 | elevated |  | ambient | peak drought | 14.97 | 1.11 | 301.73 | 285.02 | 33.74 | NA | 10.41 | 2.36 | 1.62 |
| 30 | elevated |  | reduced | peak drought | 18.42 | 1.45 | 419.59 | 282.69 | 35.13 | NA | 10.54 | 2.37 | 1.73 |
| 34 | ambient |  | reduced | peak drought | 16.18 | 0.77 | 292.66 | 281.20 | 31.43 | 28.20 | 10.65 | 2.57 | 1.68 |
| 36 | ambient |  | ambient | peak drought | 17.93 | 0.55 | 400.67 | 284.80 | 34.83 | 26.34 | 11.18 | 2.92 | 2.06 |
| 40 | elevated |  | ambient | peak drought | 26.85 | 0.86 | 281.11 | 259.53 | 40.26 | 47.00 | 9.22 | 2.26 | 1.62 |
| 42 | elevated |  | reduced | peak drought | 21.51 | 0.99 | 403.96 | 243.72 | 72.18 | 42.54 | 9.84 | 2.31 | 2.80 |
| 45 | ambient |  | reduced | peak drought | 13.76 | 0.83 | 231.89 | 302.80 | 43.54 | 26.28 | 12.75 | 3.02 | 2.34 |
| 46 | ambient |  | ambient | peak drought | 19.18 | 0.47 | 424.11 | 365.08 | 38.59 | 35.29 | 11.65 | 2.84 | 2.38 |
| 3 | elevated |  | reduced | rewetted | 29.90 | 1.67 | 106.22 | 373.81 | 33.92 | 99.60 | 13.05 | 2.65 | 1.75 |
| 4 | elevated |  | ambient | rewetted | 16.47 | 1.23 | 82.70 | 387.45 | 45.33 | 95.55 | 14.21 | 2.75 | 2.02 |
| 9 | ambient |  | ambient | rewetted | 15.02 | 1.29 | 172.27 | 359.00 | 30.67 | 81.21 | 12.54 | 2.73 | 2.49 |
| 11 | ambient |  | reduced | rewetted | 16.42 | 0.96 | 222.30 | 365.80 | 25.51 | 94.69 | 11.56 | 2.85 | 2.19 |
| 17 | elevated |  | ambient | rewetted | 23.17 | 0.88 | 126.50 | 329.02 | 37.84 | 117.94 | 11.61 | 2.59 | 1.56 |
| 18 | elevated |  | reduced | rewetted | 20.25 | 0.93 | 65.83 | 360.98 | 39.67 | 103.08 | 12.07 | 2.68 | 1.96 |
| 21 | ambient |  | ambient | rewetted | 13.03 | 0.46 | 127.75 | 334.06 | 29.16 | 50.85 | 10.33 | 2.70 | 2.11 |
| 23 | ambient |  | reduced | rewetted | 13.46 | 0.43 | 103.38 | 328.26 | 23.50 | 67.16 | 9.55 | 2.46 | 1.51 |
| 28 | elevated |  | ambient | rewetted | 21.03 | 1.31 | 77.60 | 345.66 | 25.34 | NA | 11.99 | 2.85 | 1.64 |
| 30 | elevated |  | reduced | rewetted | 13.91 | 1.34 | 46.50 | 363.74 | 30.31 | NA | 12.23 | 2.88 | 2.38 |
| 34 | ambient |  | reduced | rewetted | 22.38 | 0.82 | 93.75 | 341.11 | 21.29 | 92.31 | 14.44 | 3.37 | 1.94 |
| 36 | ambient |  | ambient | rewetted | 17.72 | 0.69 | 162.75 | 354.75 | 34.04 | 75.99 | 13.87 | 3.40 | 2.53 |
| 40 | elevated |  | ambient | rewetted | 25.16 | 1.15 | 91.52 | 363.53 | 25.87 | 101.53 | 10.14 | 2.48 | 1.72 |
| 42 | elevated |  | reduced | rewetted | 16.55 | 0.91 | 97.79 | 329.81 | 24.48 | 95.93 | 11.11 | 2.69 | 1.66 |
| 45 | ambient |  | reduced | rewetted | 14.56 | 1.50 | 94.99 | 418.73 | 28.61 | 95.28 | 14.64 | 3.45 | 2.69 |
| 46 | ambient |  | ambient | rewetted | 20.91 | 0.58 | 89.88 | 436.55 | 26.92 | 101.37 | 15.09 | 3.67 | 2.49 |
